# The Impact of COVID-19 on the Behaviors and Attitudes of Children and Adolescents: A Cross-Sectional Study

**DOI:** 10.7759/cureus.29719

**Published:** 2022-09-28

**Authors:** Swetha Annam, Maria F Fleming, Azouba Gulraiz, Muhammad Talha Zafar, Saif Khan, Princess T Oghomitse-Omene, Sana Saleemuddin, Parth Patel, Zainab Ahsan, Muhammad Saqlain S Qavi

**Affiliations:** 1 Pulmonary Medicine, Osmania Medical College, Hyderabad, IND; 2 Internal Medicine, Bhaskar Medical College, Hyderabad, IND; 3 Journal Club Leader, Forum of Artificial Intelligence in Medicine, Miami, USA; 4 General Medicine, Universidad Central de Venezuela, Caracas, VEN; 5 Medicine, California Institute of Behavioral Neurosciences & Psychology, Fairfield, USA; 6 Medicine, Liaquat National Hospital and Medical College, Karachi, PAK; 7 Hospital-based Medicine, North Manchester Hospital, Manchester, GBR; 8 Pediatrics and Neonatology, Delta State University Teaching Hospital, Abraka, NGA; 9 Pediatrics and Child Health, Convenant Community Care, Detroit, USA; 10 Family Medicine, Dr. VRK Women's Medical College, Aziznagar, USA; 11 Internal Medicine, M. P. Shah Medical College, Jamnagar, IND; 12 Medicine, CMH Lahore Medical College & Institute of Dentistry, Lahore, PAK; 13 Internal Medicine, Akhtar Saeed Medical and Dental College, Lahore, PAK

**Keywords:** mental health, child and adolescent psychiatry, adolescent, child attitudes, child behaviour, covid-19

## Abstract

Background and objective

Over the past few decades, new infectious diseases have emerged, and these have played a key role in changing behavior and lifestyle in all age groups. More recently, with the emergence of the coronavirus disease 2019 (COVID-19) pandemic, governments around the world have made unprecedented efforts to contain the epidemic by implementing quarantine measures, social distancing, and isolating infected individuals. Social behavioral adaptations (e.g., social distancing, isolation, etc.) impact children's and adolescents' lifestyle activities and lead to increased incidence of psychosocial problems, worsening of preexisting mental illness, and fears of infection, uncertainty, isolation, and stress. In light of this, this study aimed to assess the impact of COVID-19 on the behaviors and lifestyles of the children and adolescent population of Pakistan.

Methodology

A cross-sectional study was conducted involving 323 children and adolescents by targeting parents of children and adolescents in the age group of 4-18 years living in Pakistan. The study was conducted from April 2021 to September 2021. A well-designed structured questionnaire was used to collect data about the sociodemographic profile, attitudes, and behavioral factors impacted by COVID-19 in children and adolescents. SPSS Statistics version 23 (IBM, Armonk, NY) was used to enter and analyze data.

Results

Parents or caregivers of a total of 189 male and 134 female children aged between four and 18 years took part in this study. During COVID-19, the consumption of fast food and fried foods by children and adolescents increased significantly. In this study, out of 323 participants, almost all (289, 89.5%) had increased their screen time significantly. Nearly half of the total individuals experienced the feeling of depression and loneliness during the pandemic. Additionally, some children and adolescents felt fearful when leaving home. COVID-19 lockdowns have led to many changes in children's and adolescents' lifestyle habits. They reduced physical contact with others due to the fear of transmission of COVID-19. Based on our findings, the pandemic and its containment strategies have adversely affected the behaviors, lifestyles, and attitudes of children and adolescents.

Conclusion

Governments around the world have imposed social distancing during the COVID-19 pandemic, leading to adverse short-term and long-term negative mental health issues such as unhappiness, fear, worry, irritability, depressive symptoms, anxiety, and post-traumatic stress disorder (PTSD). Interventions are needed to focus on building resilience in children and adolescents, addressing their fears and concerns through better communication, encouraging routine and physical activity, and taking measures to alleviate loneliness.

## Introduction

Human behavior represents the latent and expressive capacity of various physiological, psychological, and social activities at all stages of human life; however, the earlier the habits are formed, the more likely they are to take root and flourish [1]. Over the past few decades, new infectious diseases such as severe acute respiratory syndrome (SARS), Middle East respiratory syndrome (MERS), zika, and, more recently, the coronavirus disease 2019 (COVID-19) have emerged [2]. As of May 2021, the COVID-19 pandemic has infected more than 164 million people in more than 200 countries around the world and caused more than 3,881,091 deaths, according to the World Health Organization (WHO). As a result, governments around the world have made unprecedented efforts to contain the epidemic by implementing quarantine measures, social distancing, and isolating infected individuals [3].

By the end of April 2020, an estimated 1.5 billion children (aged 5-12 years) and adolescents (aged 13-17 years) worldwide had switched to distance learning following school closures. School closures and additional social behavioral adaptations (e.g., social distancing, isolation, etc.) impacted children's and adolescents' lifestyles and activities [4]. Preliminary evidence of deep concern suggests that social restrictions needed to reduce the spread of COVID-19 have increased sedentary behavior, disrupted sleep patterns, and caused changes in lifestyles at home and outside, especially among children and adolescents [5].

A study in China found that only 17% of children achieved adequate levels of physical activity, and 66% of them were rated "inactive" during the pandemic. Canadian children reported a similar decline in physical activity [6]. The available evidence shows that 43.5% of Polish respondents said that they ate more during quarantine, and 51.8% admitted to eating snacks more frequently between meals [7]. A multicontinental survey of adolescents (n=1,047) showed that during the COVID-19 pandemic, daily sitting time increased by 28.6% and the frequency and duration of physical activity decreased by 24% and 33.5%, respectively [8]. Obese children have decreased their exercise time and increased screen and sleep time during the COVID-19-associated lockdowns [9].

The two key pillars of human civilization - social interaction and structured schedules - have been distorted by the pandemic, which has led to significant psychological effects on children and adolescents. The increased incidence of psychosocial problems, worsening of preexisting mental illnesses, and fears of infection, uncertainty, isolation, stress, and mass panic have all significantly increased due to the ongoing pandemic [10].

## Materials and methods

A web-based survey was conducted targeting the parents of children and adolescents aged 4-18 years. The data were collected from April 2021 to September 2021. An informed written consent form was developed in the English language describing the objectives of this research, and IRB approval from the Ethical Review Board of General Hospital, Lahore (00/89/20) was obtained before the data collection.

A descriptive cross-sectional study design was adopted for this research. A nonprobability, convenience sampling method was used for data collection. The Raosoft sample size calculator was used to estimate the sample size; maintaining a 5% margin of error, a 95% confidence level, and a prevalence of 31.3%, the minimum sample size of 250 was determined. We, however, conducted our study on 323 children. Parents of children and adolescents (aged 4-18 years) living in Pakistan presenting at the speech therapy clinic of our hospital were included in the study, while those who denied consent were excluded.

A standardized electronic questionnaire including queries about the sociodemographic profile, changes in eating style and behavior, changes in physical activities and screen time, lifestyle changes, and disturbances in sleep and mental health were used to collect data through a web-based survey using Google Forms. Data were entered into the SPSS Statistics version 23 (IBM, Armonk, NY). The data were coded and refined for further analysis. Results were presented in the form of frequency tables and bar/pie charts of different variables representing the research topic, and quantitative values were presented as means and standard deviations.

## Results

Sociodemographic factors

Parents of 323 children participated in this online survey. The mean age of the children was 11.2 years, with a standard deviation of ±4.2. Among them, 38.4% were between the ages of three to nine years and the rest were between the ages of 10-19; 41.5% (n=134) were females and 58.5% (n=189) were males; 58.2% (n=188) were dwelling in the metropolitan city while 41.8% (n=135) lived in small cities. The demographic factors are presented in Table 1.

**Table 1:** Sociodemographics and characteristics of participants SD: standard deviation

Variables	Values
Parental age, years, mean ±SD	39.46 ±10.4
Children age's age, years, mean ±SD	11.2 ±4.2
Gender of participants, n (%)	
Male	189 (58.5)
Female	134 (41.5)
Residence, n (%)	
Big urban center	188 (58.2)
Suburban	135 (41.8)
Level of education, n (%)	
Pre-school	70 (21.7)
Primary (till class 5)	58 (18)
Middle (6th to 8th)	23 (7.1)
Secondary (9th-10th)	33 (10.2)
College	139 (43)
Socioeconomic status, n (%)	
Lower class	4 (1.2)
Middle class	298 (92.2)
Upper class	21 (6.5)
Family status, n (%)	
Joint family	161 (49.8)
Nuclear family	162 (50.2)

Changes in eating styles and behaviors

During COVID-19, the consumption of fast food and fried foods by children and adolescents has increased significantly. According to this survey, during COVID-19, a higher consumption level of fizzy drinks has been observed. Our findings are presented in Table 2.

**Table 2:** Eating styles and behaviors of children and adolescents during the COVID-19 pandemic

Sr. no.	Eating styles and behaviors	Frequency (percentage)				
		1-2 times	3-4 times	5-6 times	Almost daily	Not routinely
1	Fast food consumption per week	122 (37.8)	52 (16.1)	8 (2.5)	28 (8.7)	113 (35)
2	Fried food consumption per week	117 (36.2)	78 (24.1)	19 (5.9)	31 (9.6)	78 (24.1)
3	Fruits consumption per week	80 (24.8)	96 (29.7)	37 (11.5)	72 (22.3)	38 (11.8)
4	Fizzy drinks consumption per week	69 (21.4)	77 (23.8)	31 (9.6)	85 (26.3)	61 (18.9)
5	Emotional eating	77 (23.8)	50 (15.5)	13 (4)	35 (10.8)	148 (45.8)

Changes in physical activities and screen time

In this study, out of 323 participants, almost all (289, 89.5%) had increased their screen time significantly. Similarly, among the 323 individuals, 52 (16.1%) spent less than two hours in front of the screen, 112 (34.7%) spent two to five hours, 93 (28.8%) spent five to eight hours, and 66 (20.4%) spent more than eight hours (Table 3).

**Table 3:** Physiological behaviors of children and adolescents during the COVID-19 pandemic

Sr. no.	Physiological behaviors	Frequency (percentage)				
		1-2 times a week	3-4 times a week	5-6 times a week	Almost daily	Not routinely
1	Participation in aerobic exercises/sports	64 (19.8)	43 (13.3)	8 (2.5)	32 (9.9)	176 (54.5)
2	Participation in household chores	66 (20.4)	51 (15.8)	5 (1.5)	55 (17)	146 (45.2)
3	Went out of the house to shop/do grocery	98 (30.3)	60 (18.6)	44 (13.6)	46 (14.2)	75 (23.3)

Mental health and sleep disturbances

Of the 323 participants, 42 said they slept for less than six hours a day, 110 said they slept six to eight hours, 133 said they slept 8-10 hours, and only 38 indicated that they slept for more than 10 hours a day. In terms of sleep quality among children and adolescents during the pandemic, it was very poor in 1.9% of the total participants, poor in 5.6%, good in 40.9%, excellent in 31.9%, while 19.8% had excellent sleep quality. Parents of 95 children stated that their children experienced unusual nightmares during sleep (Table 4).

**Table 4:** Psychological behaviors of children and adolescents during the COVID-19 pandemic

Sr. no.	Psychological behaviors	Frequency (percentage)	
		Yes	No
1	Feeling of depression among children	154 (47.7)	169 (52.3)
2	Experienced fear in leaving home	135 (41.8)	188 (58.2)
3	Felt anxious	127 (39.3)	196 (60.7)
4	Fear of getting COVID-19	170 (52.6)	153 (47.4)
5	Trouble in online learning	251 (77.4)	72 (22.6)
6	Change in attitude	195 (60.4)	128 (39.6)

Lifestyle changes

In this survey, the vast majority had recently canceled their summer vacation plans. Precautionary measures included washing hands, using hand sanitizer, wearing a mask, avoiding shaking hands, etc. Of the 323 people, nearly all washed their hands more frequently and most carried a hand sanitizer. Additionally, it was observed that almost every child engaged in increased leisure television (TV)/movie time and increased their social media usage during the COVID-19 lockdown (Table 5).

**Table 5:** Lifestyle changes observed by parents in their children during COVID-19

Sr. no.	Lifestyle changes	Frequency (percentage)	
		Yes	No
1	Limited physical contact with people	274 (84.8)	49 (15.2)
2	Avoided using healthcare facilities	219 (67.8)	104 (32.2)
3	Avoided going to public places	225 (69.7)	98 (30.3)
4	Frequency of social plans cancellation	253 (78.3)	70 (21.7)
5	Washed hands more frequently	277 (85.8)	46 (12.2)
6	Use of hand sanitizer	195 (60.4)	128 (39.6)
7	Preferred wearing a mask	297 (92)	26 (8)
8	Avoided shaking of hands	258 (79.9)	65 (20.1)
9	Stopped ordering food from outside	142 (44)	181 (56)
10	Increase in duration of watching TV/movies	297 (92)	26 (8)
11	Increase in social media use	303 (93.8)	20 (6.2)

These results indicate that COVID-19 had an impact on the lifestyle behaviors and attitudes of children and adolescents. The pandemic and the subsequent containment strategies driven by governments around the world have adversely affected the behavioral health of children and adolescents. Further studies are however needed to study the nature of these impacts in a detailed manner.

## Discussion

This study explored the experiences of children and adolescents during the COVID-19 pandemic and its impact on lifestyle attitudes and behaviors in this age group. The study gathered its findings primarily through observations by parents and caregivers. The major alterations that we observed included changes in eating behavior, physical activity, and screen time; we also noted changes in sleep, mental health, and lifestyle attitudes. A Brazilian study on food habits during the pandemic showed continued worsening in dietary patterns, with reduced intake of fruits and vegetables and increased consumption of sweets and fast food [11]. Another study in Italy showed that during the COVID-19 pandemic, 25.6% of people increased their consumption of junk food, while 29.8% reduced their consumption of fried food [12]. This disruption of healthy eating patterns can lead to obesity and malnutrition in children and adolescents. According to an Australian study (n=5,469), a significant proportion of individuals engaged in binge eating, overeating, and using food to cope during the pandemic [13]. In another UK study, more than half (53.7%) of the respondents said that their healthy perception of the food they ate in the last week had not changed compared to pre-COVID-19, while 27.9% said they were eating an unhealthy diet, and 18.4% said they ate more [14].

COVID-19 restrictions appear to have had a great impact on physical activity and behavior in children and adolescents. In an Italian study, during COVID-19 isolation, the proportion of low-active individuals increased to 39.62%, while the proportions of moderately active and highly active individuals were 29.75% and 30.63%, respectively [15]. A Canadian study of children and adolescents (n=1,472) found that during the COVID-19 pandemic, only 3.6% of children (aged 5-11 years) and 2.6% of adolescents (aged 12-17 years) carried out the suggested strategies of 60 minutes of moderate-intensity physical activity per day, which is lower than in a 2019 report that found that 12.7% met the guidelines [16].

A Chinese survey of children and adolescents (n=2,427, aged 6-17 years) reported that leisure screen time significantly increased compared to that before the COVID-19 lockdown. The total screen time increased by approximately 30 hours/week, and the time spent in the long term (≥2 hours/day) increased by 23.6% [16]. In another Canadian study, those who reduced physical activity had greater changes in screen-related sedentary behavior than those who reported increased physical activity (p=0.005) [17].

Parents reported higher levels of family stress, depression, and anxiety due to social isolation. These findings are relevant to healthy development, as adverse psychological experiences in childhood are associated with an increased risk of anxiety later in life. The public health concern is that these short-term behavioral changes in response to COVID-19 may become entrenched. In a survey conducted in Portugal, Spain, and Italy, more than half of the participants felt bored (52.2%) and a third felt lonely (37.7%), particularly among Portuguese and Italian children [18]. Another study in Turkey revealed that 54.8% of participants reported that they were afraid of getting infected by the virus, and 45.6% stated that they were afraid of spreading the virus to others [19]. In a related study in the Netherlands, 19 families with children mentioned having anxiety (25%) and following self-isolation measures in addition to government lockdown measures [20]. According to the National Coalition of Mental Illness in New York City, the number of callers seeking help with stress or anxiety increased by 60% and the average call time increased by 15 minutes in the weeks following the implementation of social distancing measures [21].

A Portuguese study showed that most patients (69.6%) reported difficulty sleeping, with waking up frequently being the most common problem during the COVID-19 lockdown [22]. Interestingly, in the Netherlands, a quarter of pre-pandemic (clinical) insomniacs had significantly improved sleep quality, while 20% of pre-pandemic good sleepers experienced poor sleep quality during lockdown measures [23]. Similarly, in a Canadian study of dream changes during the pandemic, 55% of participants (39/71) said that their dreams were more stressful, while 50.7% (36/71) said that their dreams were more stressful and vivid, while 42.2% reported experiencing more nightmares overall, with girls more likely to experience the increase in nightmares during the pandemic [24].

The rapid spread of COVID-19 to almost all regions of the world has brought about enormous health-related, environmental, economic, and social challenges. Social distancing, surgical masks, hand washing, and other precautions are considered the only way to fight the spread of the virus. All these factors can lead to changes in an individual's lifestyle. In a study conducted in Brazil, results showed that only 3.0% of the total participants reported not practicing physical and social distancing during the study period [11]. In another study, 40.9% of 4,975 adult US respondents said that they delayed or avoided any medical care, including urgent or emergency care (12.0%) and usual care (31.5%), due to concerns about COVID-19 [25].

Likewise, in a survey conducted in Hong Kong, 74.2%, 72.7%, and 59.7% of 1,501 respondents said they avoided going out, going to crowded places, and attending social gatherings of more than four people, respectively [26]. In another study conducted in Greece, one-third (28.7%) of participants said that they had canceled plans for their summer vacation, while the majority (44.9%) had not made up their minds. Only a small percentage (17.5%) believed that they will continue with their summer vacation plans with some modifications [27].

In another study conducted in Iran, the overall frequency of use of masks was 45.6% and the proportion of women using masks was significantly higher than that of men [28]. Similarly, a study conducted in Jordan showed that approximately 68.4% of contributors believed that wearing a mask could prevent infection [29] and an Indian study showed that the majority (80%) of the total participants gave an ideal answer that they avoided shaking hands during COVID-19 [30]. A major lifestyle change was the increased use of social media during the lockdown, as a study conducted in India showed an increase in the number of social media users, with 87% of people reporting an increase in their use, and 75% spending more time on social media [31].

Limitations

Like all observational studies, our study also had some limitations. Firstly, due to a strict lockdown that was in place, the study questionnaire was distributed via online platforms, including social media. The availability and use of the internet vary with different sociodemographic indicators. Therefore, this could be a hindrance to the precision of the results, especially when the ability to judge the changes in the behavior of their children also varies with the different sociodemographic settings. Secondly, our sample size of 323 also presents a challenge in adequately predicting the public perception at large and generalizing the results.

Our study, however, has been successful in addressing the specific concerns that family physicians, pediatricians, and psychiatrists would face in the future in the aftermath of the pandemic. It could also provide a framework through which more studies can be conducted to devise a strategy to cope with an upcoming "behavioral" pandemic in the future.

## Conclusions

The COVID-19 pandemic has had a major effect on the health and lifestyle behaviors of both adolescents and children. Children are less affected by COVID-19 directly but suffer indirect, potentially long-term health consequences due to changes in lifestyle, eating behavior, physical activity, sleep patterns, screen time, and mental well-being. Due to an unbalanced diet, children are at a higher risk of both obesity and malnutrition. Governments around the world have imposed social distancing, leading to adverse short-term and long-term negative mental health issues such as unhappiness, fear, worry, irritability, depressive symptoms, anxiety, and post-traumatic stress disorder (PTSD).

Parents must take care of their mental health and coping strategies and develop positive mental attitudes to support children and adolescents through this pandemic. Interventions should focus on building resilience in children and adolescents, addressing their fears and concerns through better communication, encouraging routine and physical activity, and taking measures to alleviate loneliness. Even with social distancing, social interaction is important; video conferencing, phone calls, or real-time texting may be worth considering, possibly on a daily basis.

We believe our findings on the impact of the COVID-19 pandemic on the lifestyles and behaviors of children and adolescents will encourage parents, healthcare professionals, and policymakers to put appropriate measures in place to counter them. Further studies should be conducted to investigate the nature of these impacts and to draw long-term strategies to cope with their consequences and promote physical and mental health in this age group.
